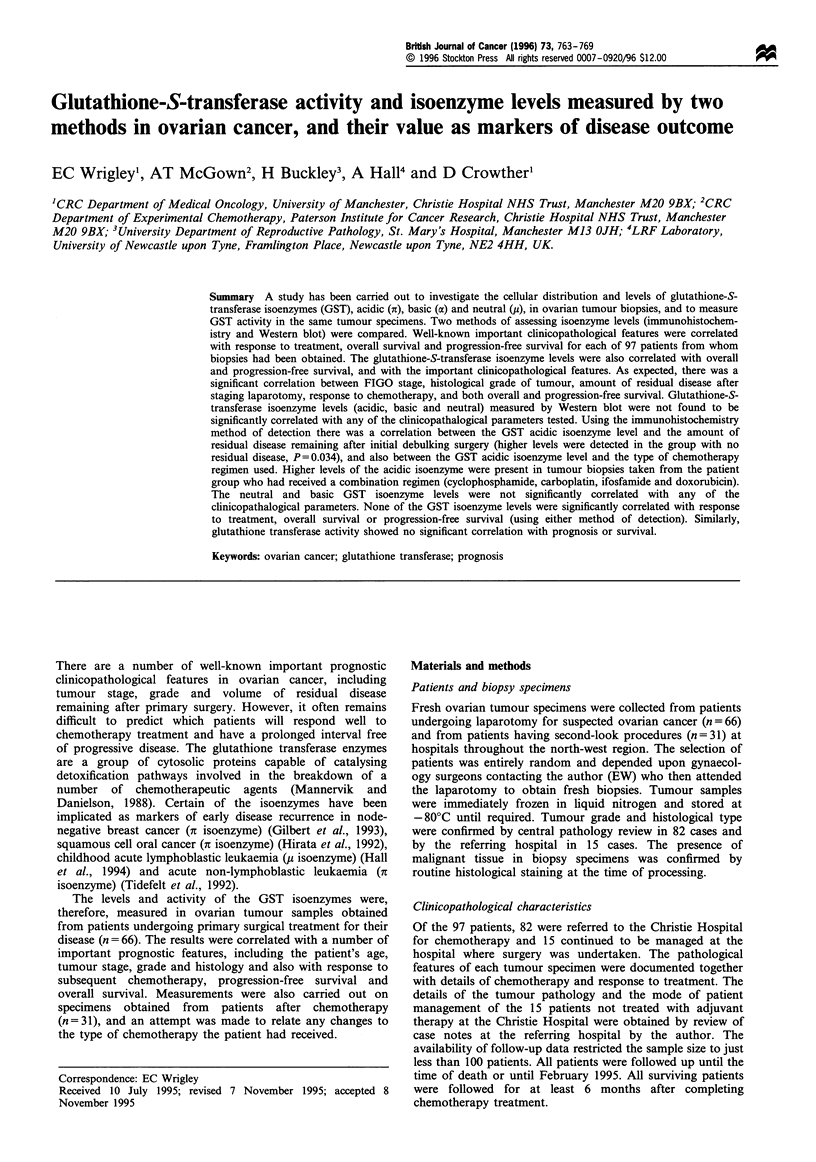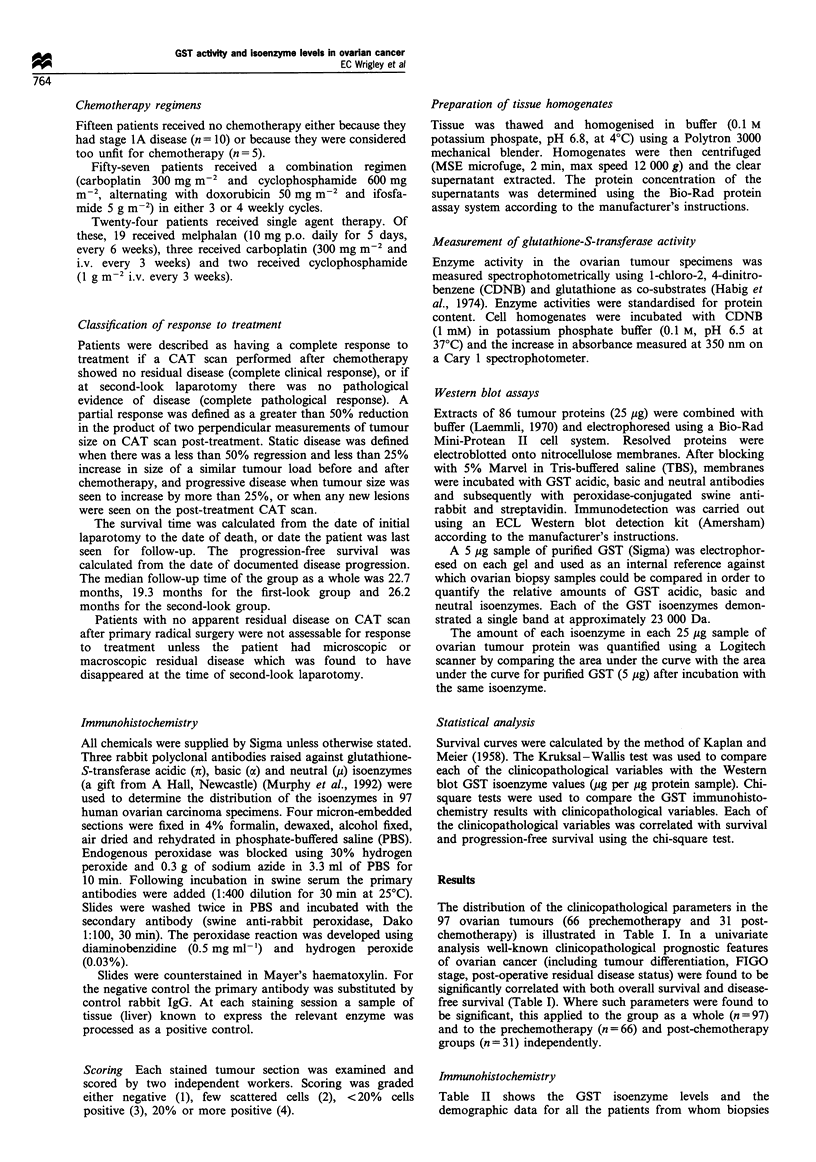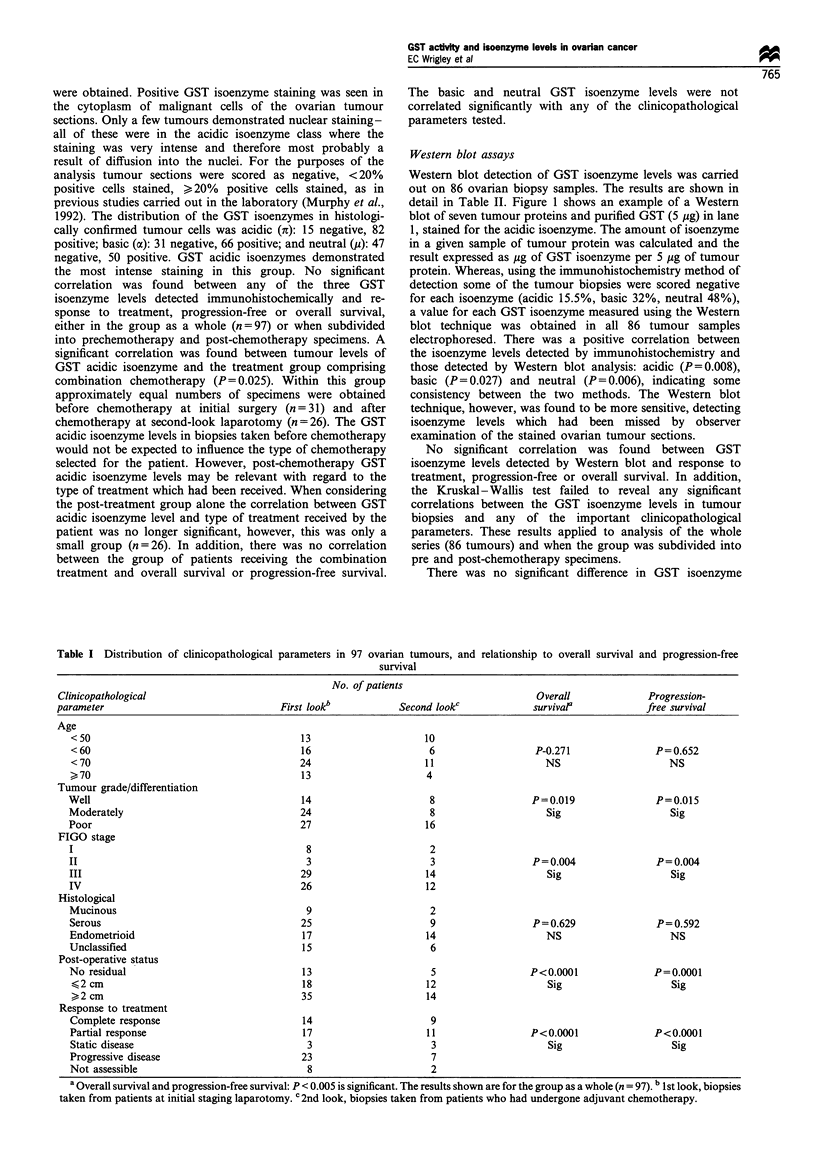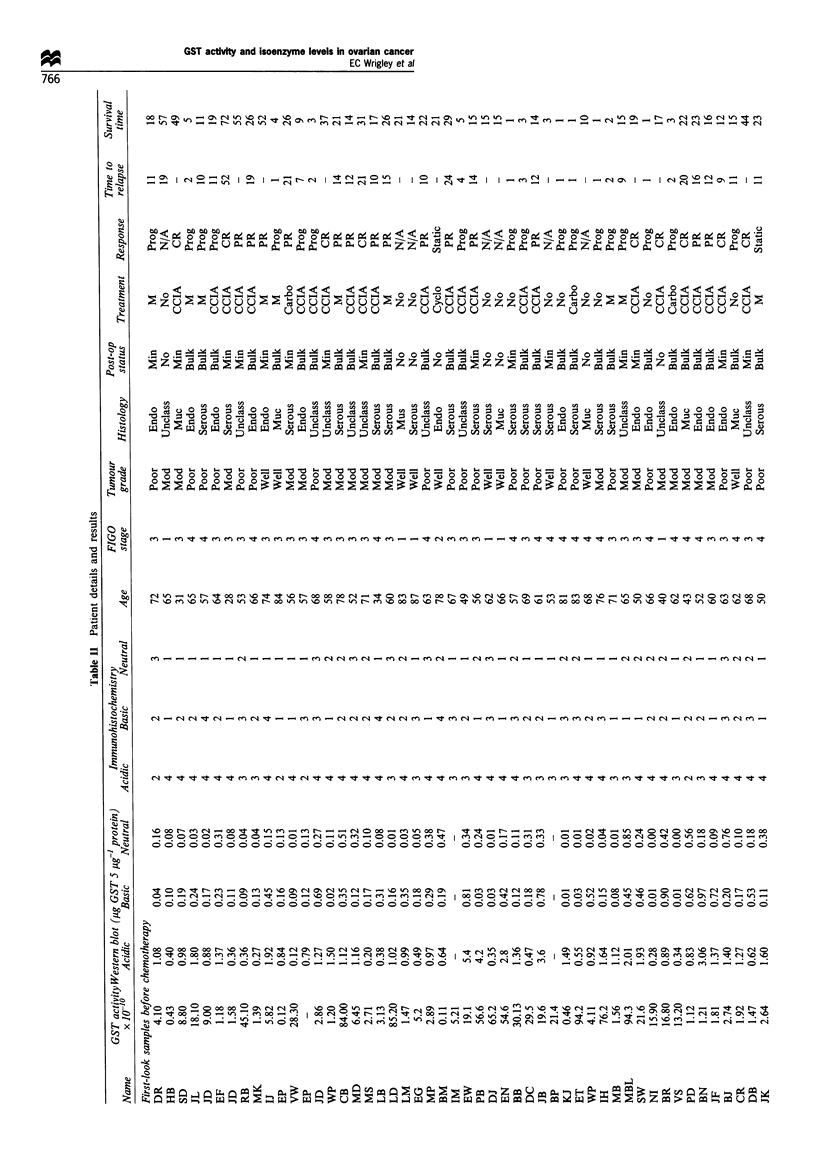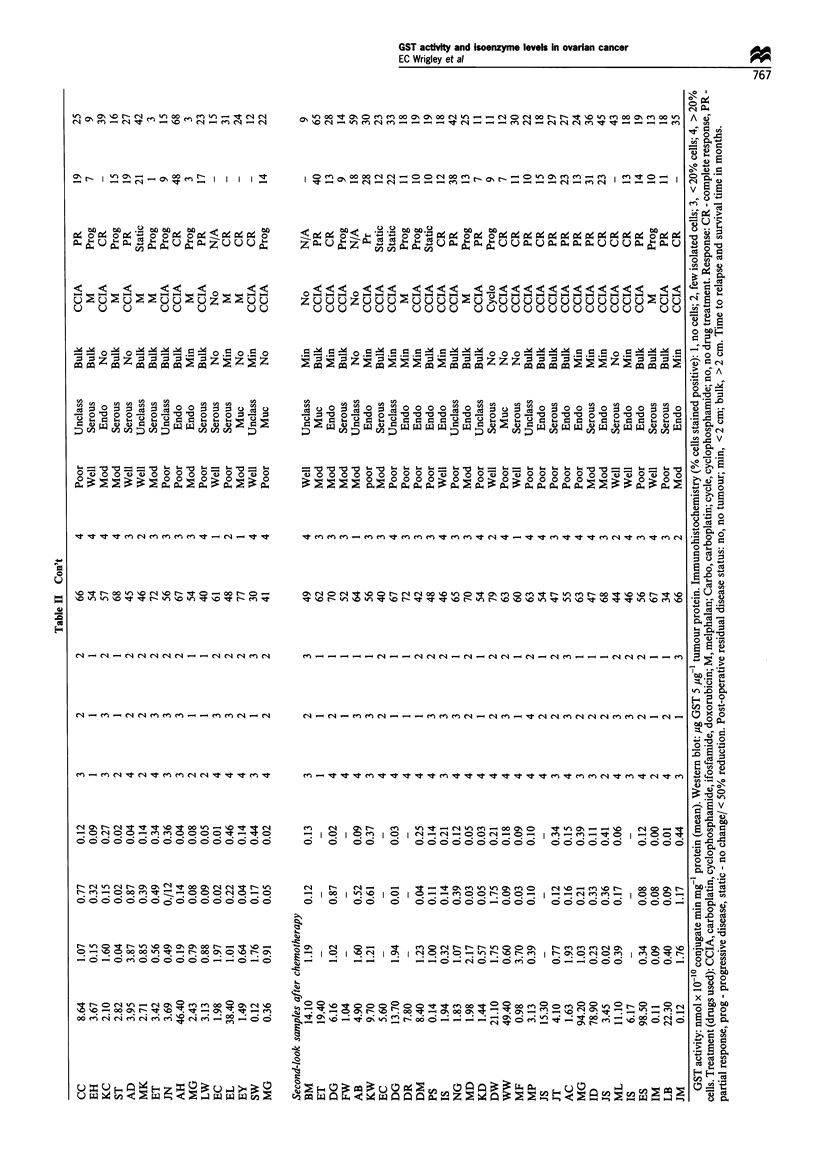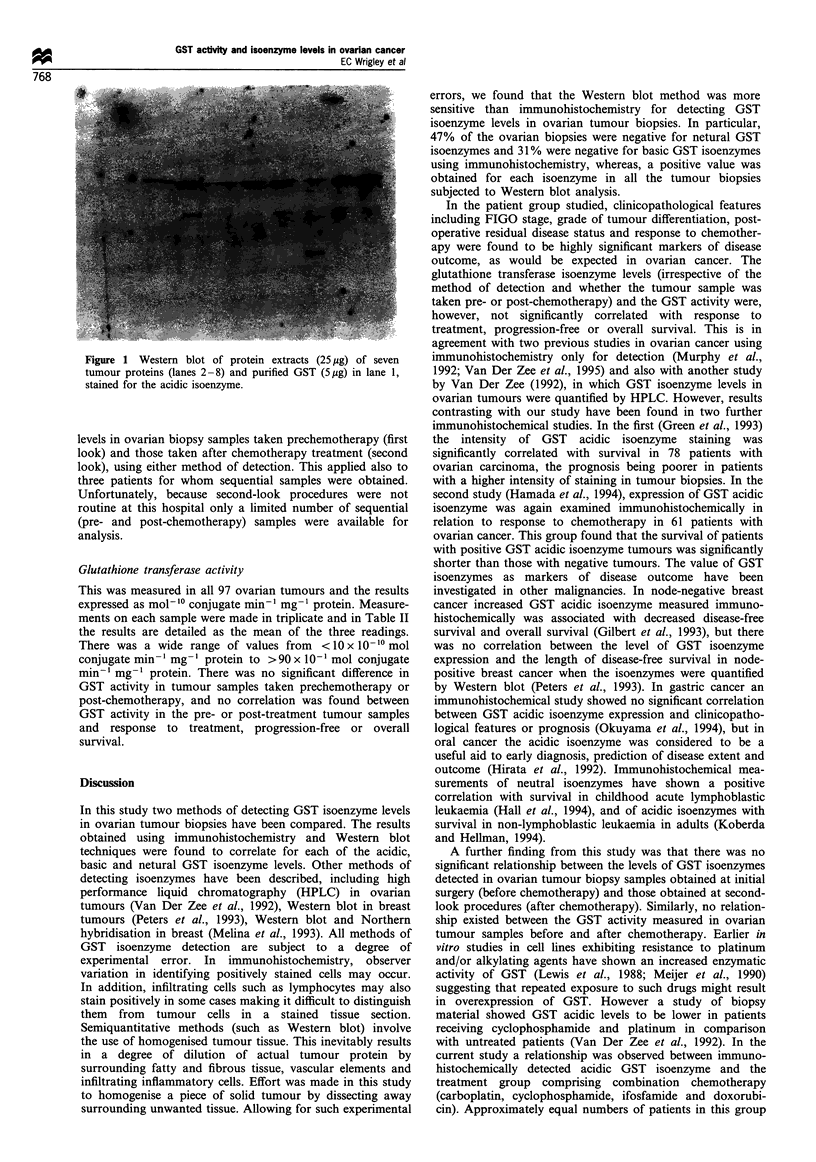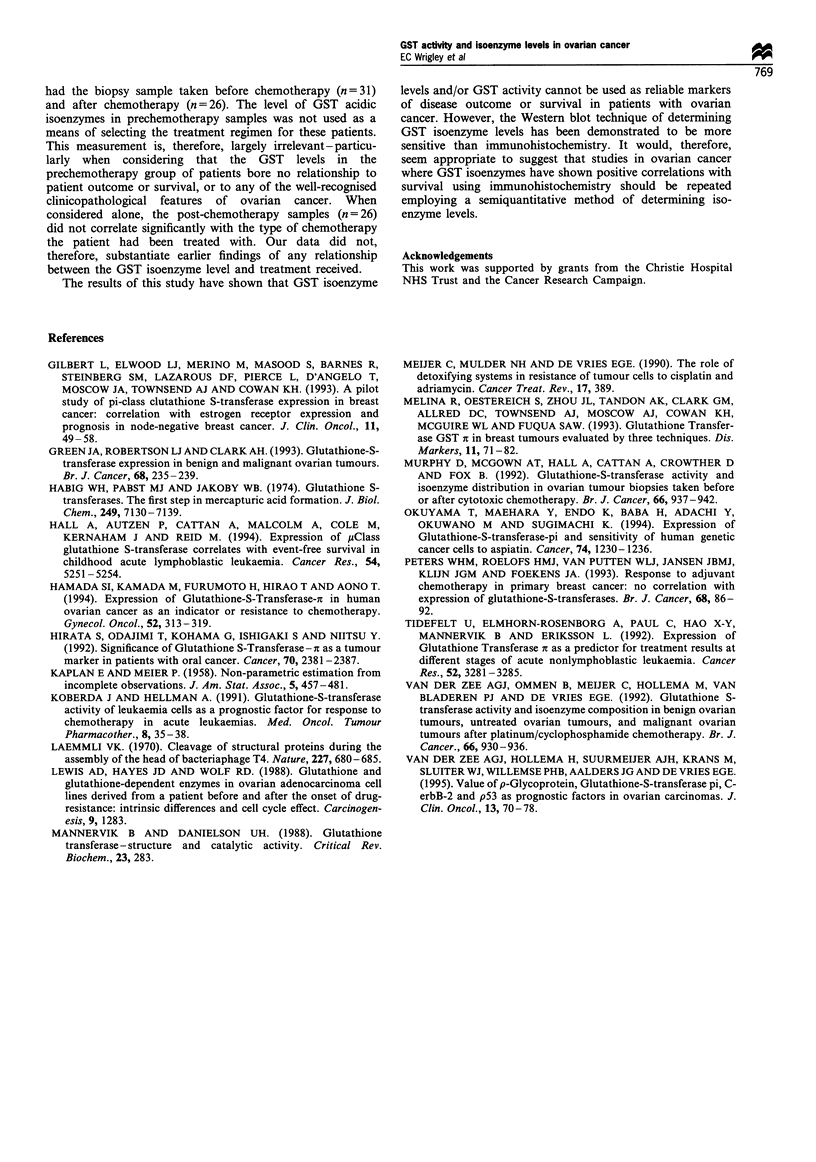# Glutathione-S-transferase activity and isoenzyme levels measured by two methods in ovarian cancer, and their value as markers of disease outcome.

**DOI:** 10.1038/bjc.1996.133

**Published:** 1996-03

**Authors:** E. C. Wrigley, A. T. McGown, H. Buckley, A. Hall, D. Crowther

**Affiliations:** CRC Department of Medical Oncology, University of Manchester, UK.

## Abstract

**Images:**


British Journal of Cancer (1996) 73, 763-769
© 1996 Stockton Press All rights reserved 0007-0920/96 $12.00

### Glutathione-S-transferase activity and isoenzyme levels measured by two methods in ovarian cancer, and their value as markers of disease outcome

EC Wrigley[1], AT McGown[2], H Buckley[3], A Hall[4] and D Crowther[1]

[1]*CRC Department of Medical Oncology, University of Manchester, Christie Hospital NHS Trust, Manchester M20 9BX;* [2]*CRC Department of Experimental Chemotherapy, Paterson Institute for Cancer Research, Christie Hospital NHS Trust, Manchester M20 9BX;* [3]*University Department of Reproductive Pathology, St. Mary's Hospital, Manchester M13 0JH;* [4]*LRF Laboratory, University of Newcastle upon Tyne, Framlington Place, Newcastle upon Tyne, NE2 4HH, UK.*

**Summary** A study has been carried out to investigate the cellular distribution and levels of glutathione-S-transferase isoenzymes (GST), acidic ($\pi$), basic ($\alpha$) and neutral ($\mu$), in ovarian tumour biopsies, and to measure GST activity in the same tumour specimens. Two methods of assessing isoenzyme levels (immunohistochemistry and Western blot) were compared. Well-known important clinicopathological features were correlated with response to treatment, overall survival and progression-free survival for each of 97 patients from whom biopsies had been obtained. The glutathione-S-transferase isoenzyme levels were also correlated with overall and progression-free survival, and with the important clinicopathological features. As expected, there was a significant correlation between FIGO stage, histological grade of tumour, amount of residual disease after staging laparotomy, response to chemotherapy, and both overall and progression-free survival. Glutathione-S-transferase isoenzyme levels (acidic, basic and neutral) measured by Western blot were not found to be significantly correlated with any of the clinicopathalogical parameters tested. Using the immunohistochemistry method of detection there was a correlation between the GST acidic isoenzyme level and the amount of residual disease remaining after initial debulking surgery (higher levels were detected in the group with no residual disease, $P=0.034$), and also between the GST acidic isoenzyme level and the type of chemotherapy regimen used. Higher levels of the acidic isoenzyme were present in tumour biopsies taken from the patient group who had received a combination regimen (cyclophosphamide, carboplatin, ifosfamide and doxorubicin). The neutral and basic GST isoenzyme levels were not significantly correlated with any of the clinicopathalogical parameters. None of the GST isoenzyme levels were significantly correlated with response to treatment, overall survival or progression-free survival (using either method of detection). Similarly, glutathione transferase activity showed no significant correlation with prognosis or survival.

**Keywords:** ovarian cancer; glutathione transferase; prognosis

There are a number of well-known important prognostic clinicopathological features in ovarian cancer, including tumour stage, grade and volume of residual disease remaining after primary surgery. However, it often remains difficult to predict which patients will respond well to chemotherapy treatment and have a prolonged interval free of progressive disease. The glutathione transferase enzymes are a group of cytosolic proteins capable of catalysing detoxification pathways involved in the breakdown of a number of chemotherapeutic agents (Mannervik and Danielson, 1988). Certain of the isoenzymes have been implicated as markers of early disease recurrence in node-negative breast cancer ($\pi$ isoenzyme) (Gilbert *et al.*, 1993), squamous cell oral cancer ($\pi$ isoenzyme) (Hirata *et al.*, 1992), childhood acute lymphoblastic leukaemia ($\mu$ isoenzyme) (Hall *et al.*, 1994) and acute non-lymphoblastic leukaemia ($\pi$ isoenzyme) (Tidefelt *et al.*, 1992).

The levels and activity of the GST isoenzymes were, therefore, measured in ovarian tumour samples obtained from patients undergoing primary surgical treatment for their disease ($n=66$). The results were correlated with a number of important prognostic features, including the patient's age, tumour stage, grade and histology and also with response to subsequent chemotherapy, progression-free survival and overall survival. Measurements were also carried out on specimens obtained from patients after chemotherapy ($n=31$), and an attempt was made to relate any changes to the type of chemotherapy the patient had received.

Correspondence: EC Wrigley
Received 10 July 1995; revised 7 November 1995; accepted 8 November 1995

### Materials and methods

### *Patients and biopsy specimens*

Fresh ovarian tumour specimens were collected from patients undergoing laparotomy for suspected ovarian cancer ($n=66$) and from patients having second-look procedures ($n=31$) at hospitals throughout the north-west region. The selection of patients was entirely random and depended upon gynaecology surgeons contacting the author (EW) who then attended the laparotomy to obtain fresh biopsies. Tumour samples were immediately frozen in liquid nitrogen and stored at $-80°C$ until required. Tumour grade and histological type were confirmed by central pathology review in 82 cases and by the referring hospital in 15 cases. The presence of malignant tissue in biopsy specimens was confirmed by routine histological staining at the time of processing.

### *Clinicopathological characteristics*

Of the 97 patients, 82 were referred to the Christie Hospital for chemotherapy and 15 continued to be managed at the hospital where surgery was undertaken. The pathological features of each tumour specimen were documented together with details of chemotherapy and response to treatment. The details of the tumour pathology and the mode of patient management of the 15 patients not treated with adjuvant therapy at the Christie Hospital were obtained by review of case notes at the referring hospital by the author. The availability of follow-up data restricted the sample size to just less than 100 patients. All patients were followed up until the time of death or until February 1995. All surviving patients were followed for at least 6 months after completing chemotherapy treatment.

*Chemotherapy regimens*

Fifteen patients received no chemotherapy either because they had stage 1A disease ($n = 10$) or because they were considered too unfit for chemotherapy ($n = 5$).

Fifty-seven patients received a combination regimen (carboplatin 300 mg $m^{-2}$ and cyclophosphamide 600 mg $m^{-2}$, alternating with doxorubicin 50 mg $m^{-2}$ and ifosfamide 5 g $m^{-2}$) in either 3 or 4 weekly cycles.

Twenty-four patients received single agent therapy. Of these, 19 received melphalan (10 mg p.o. daily for 5 days, every 6 weeks), three received carboplatin (300 mg $m^{-2}$ and i.v. every 3 weeks) and two received cyclophosphamide (1 g $m^{-2}$ i.v. every 3 weeks).

*Classification of response to treatment*

Patients were described as having a complete response to treatment if a CAT scan performed after chemotherapy showed no residual disease (complete clinical response), or if at second-look laparotomy there was no pathological evidence of disease (complete pathological response). A partial response was defined as a greater than 50% reduction in the product of two perpendicular measurements of tumour size on CAT scan post-treatment. Static disease was defined when there was a less than 50% regression and less than 25% increase in size of a similar tumour load before and after chemotherapy, and progressive disease when tumour size was seen to increase by more than 25%, or when any new lesions were seen on the post-treatment CAT scan.

The survival time was calculated from the date of initial laparotomy to the date of death, or date the patient was last seen for follow-up. The progression-free survival was calculated from the date of documented disease progression. The median follow-up time of the group as a whole was 22.7 months, 19.3 months for the first-look group and 26.2 months for the second-look group.

Patients with no apparent residual disease on CAT scan after primary radical surgery were not assessable for response to treatment unless the patient had microscopic or macroscopic residual disease which was found to have disappeared at the time of second-look laparotomy.

*Immunohistochemistry*

All chemicals were supplied by Sigma unless otherwise stated. Three rabbit polyclonal antibodies raised against glutathione-*S*-transferase acidic ($\pi$), basic ($\alpha$) and neutral ($\mu$) isoenzymes (a gift from A Hall, Newcastle) (Murphy *et al.*, 1992) were used to determine the distribution of the isoenzymes in 97 human ovarian carcinoma specimens. Four micron-embedded sections were fixed in 4% formalin, dewaxed, alcohol fixed, air dried and rehydrated in phosphate-buffered saline (PBS). Endogenous peroxidase was blocked using 30% hydrogen peroxide and 0.3 g of sodium azide in 3.3 ml of PBS for 10 min. Following incubation in swine serum the primary antibodies were added (1:400 dilution for 30 min at 25°C). Slides were washed twice in PBS and incubated with the secondary antibody (swine anti-rabbit peroxidase, Dako 1:100, 30 min). The peroxidase reaction was developed using diaminobenzidine (0.5 mg $ml^{-1}$) and hydrogen peroxide (0.03%).

Slides were counterstained in Mayer's haematoxylin. For the negative control the primary antibody was substituted by control rabbit IgG. At each staining session a sample of tissue (liver) known to express the relevant enzyme was processed as a positive control.

*Scoring* Each stained tumour section was examined and scored by two independent workers. Scoring was graded either negative (1), few scattered cells (2), <20% cells positive (3), 20% or more positive (4).

*Preparation of tissue homogenates*

Tissue was thawed and homogenised in buffer (0.1 M potassium phospate, pH 6.8, at 4°C) using a Polytron 3000 mechanical blender. Homogenates were then centrifuged (MSE microfuge, 2 min, max speed 12 000 ***g***) and the clear supernatant extracted. The protein concentration of the supernatants was determined using the Bio-Rad protein assay system according to the manufacturer's instructions.

*Measurement of glutathione-S-transferase activity*

Enzyme activity in the ovarian tumour specimens was measured spectrophotometrically using 1-chloro-2, 4-dinitrobenzene (CDNB) and glutathione as co-substrates (Habig *et al.*, 1974). Enzyme activities were standardised for protein content. Cell homogenates were incubated with CDNB (1 mM) in potassium phosphate buffer (0.1 M, pH 6.5 at 37°C) and the increase in absorbance measured at 350 nm on a Cary 1 spectrophotometer.

*Western blot assays*

Extracts of 86 tumour proteins (25 $\mu$g) were combined with buffer (Laemmli, 1970) and electrophoresed using a Bio-Rad Mini-Protean II cell system. Resolved proteins were electroblotted onto nitrocellulose membranes. After blocking with 5% Marvel in Tris-buffered saline (TBS), membranes were incubated with GST acidic, basic and neutral antibodies and subsequently with peroxidase-conjugated swine anti-rabbit and streptavidin. Immunodetection was carried out using an ECL Western blot detection kit (Amersham) according to the manufacturer's instructions.

A 5 $\mu$g sample of purified GST (Sigma) was electrophoresed on each gel and used as an internal reference against which ovarian biopsy samples could be compared in order to quantify the relative amounts of GST acidic, basic and neutral isoenzymes. Each of the GST isoenzymes demonstrated a single band at approximately 23 000 Da.

The amount of each isoenzyme in each 25 $\mu$g sample of ovarian tumour protein was quantified using a Logitech scanner by comparing the area under the curve with the area under the curve for purified GST (5 $\mu$g) after incubation with the same isoenzyme.

*Statistical analysis*

Survival curves were calculated by the method of Kaplan and Meier (1958). The Kruksal–Wallis test was used to compare each of the clinicopathological variables with the Western blot GST isoenzyme values ($\mu$g per $\mu$g protein sample). Chi-square tests were used to compare the GST immunohistochemistry results with clinicopathological variables. Each of the clinicopathological variables was correlated with survival and progression-free survival using the chi-square test.

### Results

The distribution of the clinicopathological parameters in the 97 ovarian tumours (66 prechemotherapy and 31 post-chemotherapy) is illustrated in Table I. In a univariate analysis well-known clinicopathological prognostic features of ovarian cancer (including tumour differentiation, FIGO stage, post-operative residual disease status) were found to be significantly correlated with both overall survival and disease-free survival (Table I). Where such parameters were found to be significant, this applied to the group as a whole ($n = 97$) and to the prechemotherapy ($n = 66$) and post-chemotherapy groups ($n = 31$) independently.

*Immunohistochemistry*

Table II shows the GST isoenzyme levels and the demographic data for all the patients from whom biopsies were obtained. Positive GST isoenzyme staining was seen in the cytoplasm of malignant cells of the ovarian tumour sections. Only a few tumours demonstrated nuclear staining – all of these were in the acidic isoenzyme class where the staining was very intense and therefore most probably a result of diffusion into the nuclei. For the purposes of the analysis tumour sections were scored as negative, <20% positive cells stained, ⩾20% positive cells stained, as in previous studies carried out in the laboratory (Murphy *et al.*, 1992). The distribution of the GST isoenzymes in histologically confirmed tumour cells was acidic ($\pi$): 15 negative, 82 positive; basic ($\alpha$): 31 negative, 66 positive; and neutral ($\mu$): 47 negative, 50 positive. GST acidic isoenzymes demonstrated the most intense staining in this group. No significant correlation was found between any of the three GST isoenzyme levels detected immunohistochemically and response to treatment, progression-free or overall survival, either in the group as a whole ($n=97$) or when subdivided into prechemotherapy and post-chemotherapy specimens. A significant correlation was found between tumour levels of GST acidic isoenzyme and the treatment group comprising combination chemotherapy ($P=0.025$). Within this group approximately equal numbers of specimens were obtained before chemotherapy at initial surgery ($n=31$) and after chemotherapy at second-look laparotomy ($n=26$). The GST acidic isoenzyme levels in biopsies taken before chemotherapy would not be expected to influence the type of chemotherapy selected for the patient. However, post-chemotherapy GST acidic isoenzyme levels may be relevant with regard to the type of treatment which had been received. When considering the post-treatment group alone the correlation between GST acidic isoenzyme level and type of treatment received by the patient was no longer significant, however, this was only a small group ($n=26$). In addition, there was no correlation between the group of patients receiving the combination treatment and overall survival or progression-free survival. The basic and neutral GST isoenzyme levels were not correlated significantly with any of the clinicopathological parameters tested.

### *Western blot assays*

Western blot detection of GST isoenzyme levels was carried out on 86 ovarian biopsy samples. The results are shown in detail in Table II. Figure 1 shows an example of a Western blot of seven tumour proteins and purified GST (5 $\mu$g) in lane 1, stained for the acidic isoenzyme. The amount of isoenzyme in a given sample of tumour protein was calculated and the result expressed as $\mu$g of GST isoenzyme per 5 $\mu$g of tumour protein. Whereas, using the immunohistochemistry method of detection some of the tumour biopsies were scored negative for each isoenzyme (acidic 15.5%, basic 32%, neutral 48%), a value for each GST isoenzyme measured using the Western blot technique was obtained in all 86 tumour samples electrophoresed. There was a positive correlation between the isoenzyme levels detected by immunohistochemistry and those detected by Western blot analysis: acidic ($P=0.008$), basic ($P=0.027$) and neutral ($P=0.006$), indicating some consistency between the two methods. The Western blot technique, however, was found to be more sensitive, detecting isoenzyme levels which had been missed by observer examination of the stained ovarian tumour sections.

No significant correlation was found between GST isoenzyme levels detected by Western blot and response to treatment, progression-free or overall survival. In addition, the Kruskal–Wallis test failed to reveal any significant correlations between the GST isoenzyme levels in tumour biopsies and any of the important clinicopathological parameters. These results applied to analysis of the whole series (86 tumours) and when the group was subdivided into pre and post-chemotherapy specimens.

There was no significant difference in GST isoenzyme

**Table I** Distribution of clinicopathological parameters in 97 ovarian tumours, and relationship to overall survival and progression-free survival

| *Clinicopathological parameter* | *No. of patients* First look[b] | Second look[c] | *Overall survival*[a] | *Progression-free survival* |
|---|---|---|---|---|
| Age | | | | |
| <50 | 13 | 10 | | |
| <60 | 16 | 6 | P-0.271 | P=0.652 |
| <70 | 24 | 11 | NS | NS |
| ⩾70 | 13 | 4 | | |
| Tumour grade/differentiation | | | | |
| Well | 14 | 8 | P=0.019 | P=0.015 |
| Moderately | 24 | 8 | Sig | Sig |
| Poor | 27 | 16 | | |
| FIGO stage | | | | |
| I | 8 | 2 | | |
| II | 3 | 3 | P=0.004 | P=0.004 |
| III | 29 | 14 | Sig | Sig |
| IV | 26 | 12 | | |
| Histological | | | | |
| Mucinous | 9 | 2 | | |
| Serous | 25 | 9 | P=0.629 | P=0.592 |
| Endometrioid | 17 | 14 | NS | NS |
| Unclassified | 15 | 6 | | |
| Post-operative status | | | | |
| No residual | 13 | 5 | P<0.0001 | P=0.0001 |
| ⩽2 cm | 18 | 12 | Sig | Sig |
| ⩾2 cm | 35 | 14 | | |
| Response to treatment | | | | |
| Complete response | 14 | 9 | | |
| Partial response | 17 | 11 | P<0.0001 | P<0.0001 |
| Static disease | 3 | 3 | Sig | Sig |
| Progressive disease | 23 | 7 | | |
| Not assessible | 8 | 2 | | |

[a] Overall survival and progression-free survival: $P<0.005$ is significant. The results shown are for the group as a whole ($n=97$). [b] 1st look, biopsies taken from patients at initial staging laparotomy. [c] 2nd look, biopsies taken from patients who had undergone adjuvant chemotherapy.

**Table II** Patient details and results

| *Name* | *GST activity × $10^{-10}$* | *Western blot (µg GST 5 µg$^{-1}$ protein)* Acidic | Basic | Neutral | *Immunohistochemistry* Acidic | Basic | Neutral | *Age* | *FIGO stage* | *Tumour grade* | *Histology* | *Post-op status* | *Treatment* | *Response* | *Time to relapse* | *Survival time* |
|---|---|---|---|---|---|---|---|---|---|---|---|---|---|---|---|---|
| *First-look samples before chemotherapy* | | | | | | | | | | | | | | | | |
| DR | 4.10 | 1.08 | 0.04 | 0.16 | 2 | 2 | 3 | 72 | 3 | Poor | Endo | Min | M | Prog | 11 | 18 |
| HB | 0.43 | 0.40 | 0.10 | 0.08 | 4 | 1 | 1 | 65 | 1 | Mod | Unclass | No | No | N/A | 19 | 57 |
| SD | 8.80 | 0.98 | 0.19 | 0.07 | 4 | 2 | 1 | 31 | 3 | Mod | Muc | Min | CCIA | CR | – | 49 |
| JL | 18.10 | 1.80 | 0.24 | 0.03 | 4 | 2 | 1 | 65 | 4 | Poor | Endo | Bulk | M | Prog | 2 | 5 |
| JD | 9.00 | 0.88 | 0.17 | 0.02 | 4 | 4 | 1 | 57 | 4 | Poor | Serous | Bulk | M | Prog | 10 | 11 |
| EF | 1.18 | 1.37 | 0.23 | 0.31 | 4 | 2 | 1 | 64 | 3 | Poor | Endo | Bulk | CCIA | Prog | 11 | 19 |
| JD | 1.58 | 0.36 | 0.11 | 0.08 | 4 | 1 | 1 | 28 | 3 | Mod | Serous | Min | CCIA | CR | 52 | 72 |
| RB | 45.10 | 0.36 | 0.09 | 0.04 | 3 | 3 | 2 | 53 | 3 | Poor | Unclass | Min | CCIA | PR | – | 55 |
| MK | 1.39 | 0.27 | 0.13 | 0.04 | 3 | 2 | 1 | 66 | 4 | Poor | Endo | Bulk | CCIA | PR | 19 | 26 |
| IJ | 5.82 | 1.92 | 0.45 | 0.15 | 4 | 4 | 1 | 74 | 3 | Well | Endo | Min | M | PR | – | 52 |
| EP | 0.12 | 0.84 | 0.16 | 0.13 | 2 | 1 | 1 | 84 | 3 | Well | Muc | Bulk | M | Prog | 1 | 4 |
| VW | 28.30 | 0.12 | 0.09 | 0.01 | 4 | 1 | 1 | 56 | 3 | Mod | Serous | Min | Carbo | PR | 21 | 26 |
| EP | – | 0.79 | 0.12 | 0.13 | 2 | 3 | 1 | 57 | 3 | Mod | Endo | Bulk | CCIA | Prog | 7 | 9 |
| JD | 2.86 | 1.27 | 0.69 | 0.27 | 4 | 3 | 3 | 68 | 4 | Poor | Unclass | Bulk | CCIA | Prog | 2 | 3 |
| WP | 1.20 | 1.50 | 0.02 | 0.11 | 4 | 1 | 2 | 58 | 3 | Mod | Unclass | Min | CCIA | CR | – | 37 |
| CB | 84.00 | 1.12 | 0.35 | 0.51 | 4 | 2 | 2 | 78 | 3 | Mod | Serous | Bulk | M | PR | 14 | 21 |
| MD | 6.45 | 1.16 | 0.12 | 0.32 | 4 | 2 | 3 | 52 | 3 | Mod | Unclass | Bulk | CCIA | PR | 12 | 14 |
| MS | 2.71 | 0.20 | 0.17 | 0.10 | 4 | 2 | 2 | 71 | 3 | Mod | Unclass | Min | CCIA | CR | 21 | 31 |
| LB | 3.13 | 0.38 | 0.31 | 0.08 | 4 | 4 | 1 | 34 | 4 | Mod | Serous | Bulk | CCIA | PR | 10 | 17 |
| LD | 85.20 | 1.02 | 0.16 | 0.01 | 3 | 2 | 3 | 60 | 3 | Mod | Serous | Bulk | M | PR | 15 | 26 |
| LM | 1.47 | 0.99 | 0.35 | 0.03 | 4 | 2 | 2 | 83 | 1 | Well | Mus | No | No | N/A | – | 21 |
| EG | 5.2 | 0.49 | 0.18 | 0.05 | 3 | 3 | 1 | 87 | 1 | Well | Serous | No | No | N/A | – | 14 |
| MP | 2.89 | 0.97 | 0.29 | 0.38 | 4 | 1 | 3 | 63 | 4 | Poor | Unclass | Bulk | CCIA | PR | 10 | 22 |
| BM | 0.11 | 0.64 | 0.19 | 0.47 | 4 | 4 | 2 | 78 | 2 | Well | Endo | No | Cyclo | Static | – | 21 |
| IM | 5.21 | – | – | – | 3 | 3 | 1 | 67 | 3 | Poor | Serous | Bulk | CCIA | PR | 24 | 29 |
| EW | 19.1 | 5.4 | 0.81 | 0.34 | 3 | 2 | 1 | 49 | 3 | Poor | Unclass | Bulk | CCIA | Prog | 4 | 5 |
| PB | 56.6 | 4.2 | 0.03 | 0.24 | 4 | 1 | 2 | 56 | 3 | Poor | Serous | Min | CCIA | PR | 14 | 15 |
| DJ | 65.2 | 0.35 | 0.03 | 0.01 | 4 | 3 | 3 | 62 | 1 | Well | Serous | No | No | N/A | – | 15 |
| EN | 54.6 | 2.8 | 0.42 | 0.17 | 4 | 1 | 1 | 66 | 1 | Well | Muc | No | No | N/A | – | 15 |
| BB | 30.13 | 1.36 | 0.12 | 0.11 | 4 | 3 | 2 | 57 | 4 | Poor | Serous | Min | No | Prog | 1 | 1 |
| DC | 29.5 | 0.47 | 0.18 | 0.31 | 3 | 2 | 1 | 69 | 3 | Poor | Serous | Bulk | CCIA | Prog | 3 | 3 |
| JB | 19.6 | 3.6 | 0.78 | 0.33 | 3 | 2 | 1 | 61 | 4 | Poor | Serous | Bulk | CCIA | PR | 12 | 14 |
| BP | 21.4 | – | – | – | 3 | 1 | 1 | 53 | 4 | Well | Serous | Min | No | N/A | – | 3 |
| KJ | 0.46 | 1.49 | 0.01 | 0.01 | 3 | 3 | 2 | 81 | 4 | Poor | Endo | Bulk | No | Prog | 1 | 1 |
| ET | 94.2 | 0.55 | 0.03 | 0.01 | 4 | 3 | 2 | 83 | 4 | Poor | Serous | Bulk | Carbo | Prog | 1 | 1 |
| WP | 4.11 | 0.92 | 0.52 | 0.02 | 4 | 2 | 1 | 68 | 4 | Well | Muc | No | No | N/A | – | 10 |
| IH | 76.2 | 1.64 | 0.15 | 0.04 | 4 | 3 | 1 | 76 | 4 | Mod | Serous | Bulk | No | Prog | 1 | 1 |
| MB | 1.56 | 1.12 | 0.08 | 0.01 | 3 | 1 | 1 | 71 | 3 | Poor | Serous | Bulk | M | Prog | 2 | 2 |
| MBL | 94.3 | 2.01 | 0.45 | 0.85 | 3 | 1 | 2 | 65 | 3 | Mod | Unclass | Min | M | Prog | 9 | 15 |
| SW | 21.6 | 1.93 | 0.46 | 0.24 | 4 | 1 | 2 | 50 | 3 | Mod | Endo | Min | CCIA | CR | – | 19 |
| NI | 15.90 | 0.28 | 0.01 | 0.00 | 4 | 2 | 2 | 66 | 4 | Poor | Endo | Bulk | No | Prog | 1 | 1 |
| BR | 16.80 | 0.89 | 0.90 | 0.42 | 4 | 2 | 2 | 40 | 1 | Mod | Unclass | No | CCIA | CR | – | 17 |
| VS | 13.20 | 0.34 | 0.01 | 0.00 | 3 | 1 | 1 | 62 | 4 | Mod | Endo | Bulk | Carbo | Prog | 2 | 3 |
| PD | 1.12 | 0.83 | 0.62 | 0.56 | 2 | 2 | 2 | 43 | 4 | Mod | Muc | Bulk | CCIA | CR | 20 | 22 |
| BN | 1.21 | 3.06 | 0.97 | 0.18 | 3 | 2 | 1 | 52 | 4 | Mod | Endo | Bulk | CCIA | PR | 16 | 23 |
| JF | 1.81 | 1.37 | 0.72 | 0.09 | 4 | 1 | 1 | 60 | 3 | Mod | Endo | Bulk | CCIA | PR | 12 | 16 |
| BJ | 2.74 | 1.40 | 0.20 | 0.76 | 4 | 3 | 3 | 63 | 3 | Poor | Endo | Min | CCIA | CR | 9 | 12 |
| CR | 1.92 | 1.27 | 0.17 | 0.10 | 4 | 2 | 2 | 62 | 4 | Well | Muc | Bulk | No | Prog | 11 | 15 |
| DB | 1.47 | 0.62 | 0.53 | 0.18 | 4 | 3 | 2 | 68 | 3 | Poor | Unclass | Min | CCIA | CR | – | 44 |
| JK | 2.64 | 1.60 | 0.11 | 0.38 | 4 | 1 | 1 | 50 | 4 | Poor | Serous | Bulk | M | Static | 11 | 23 |

Table II Con't

| | | | | | | | | | | | | | | | | |
|---|---|---|---|---|---|---|---|---|---|---|---|---|---|---|---|---|
| CC | 8.64 | 1.07 | 0.77 | 0.12 | 3 | 2 | 2 | 66 | 4 | Poor | Unclass | Bulk | CCIA | PR | 19 | 25 |
| EH | 3.67 | 0.15 | 0.32 | 0.09 | 1 | 1 | 1 | 54 | 4 | Well | Serous | Bulk | M | Prog | 7 | 9 |
| KC | 2.10 | 1.60 | 0.15 | 0.27 | 3 | 3 | 2 | 57 | 4 | Mod | Endo | No | CCIA | CR | – | 39 |
| ST | 2.82 | 0.04 | 0.02 | 0.02 | 2 | 1 | 1 | 68 | 4 | Mod | Serous | Bulk | M | Prog | 15 | 16 |
| AD | 3.95 | 3.87 | 0.87 | 0.04 | 4 | 2 | 2 | 45 | 3 | Well | Serous | No | CCIA | PR | 19 | 27 |
| MK | 2.71 | 0.85 | 0.39 | 0.14 | 2 | 2 | 2 | 46 | 2 | Well | Unclass | Bulk | M | Static | 21 | 42 |
| ET | 3.42 | 0.56 | 0.49 | 0.34 | 4 | 3 | 2 | 72 | 3 | Mod | Serous | Bulk | M | Prog | 1 | 3 |
| JN | 3.69 | 0.49 | 0./12 | 0.36 | 3 | 3 | 2 | 56 | 3 | Poor | Unclass | Bulk | CCIA | Prog | 9 | 15 |
| AH | 46.40 | 0.19 | 0.14 | 0.04 | 3 | 3 | 2 | 67 | 3 | Poor | Endo | Bulk | CCIA | CR | 48 | 68 |
| MG | 2.43 | 0.79 | 0.08 | 0.08 | 2 | 1 | 1 | 54 | 3 | Mod | Endo | Min | M | Prog | 3 | 3 |
| LW | 3.13 | 0.88 | 0.09 | 0.05 | 2 | 1 | 1 | 40 | 4 | Poor | Serous | Bulk | CCIA | PR | 17 | 23 |
| EC | 1.98 | 1.97 | 0.02 | 0.01 | 4 | 3 | 2 | 61 | 1 | Well | Serous | No | No | N/A | – | 15 |
| EL | 38.40 | 1.01 | 0.22 | 0.46 | 4 | 3 | 2 | 48 | 2 | Poor | Serous | Min | M | CR | – | 31 |
| EY | 1.49 | 0.64 | 0.04 | 0.14 | 4 | 2 | 2 | 77 | 1 | Mod | Muc | No | M | CR | – | 24 |
| SW | 0.12 | 1.76 | 0.17 | 0.44 | 3 | 1 | 3 | 30 | 4 | Well | Unclass | Min | CCIA | CR | – | 12 |
| MG | 0.36 | 0.91 | 0.05 | 0.02 | 4 | 2 | 2 | 41 | 4 | Poor | Muc | No | CCIA | Prog | 14 | 22 |
| *Second-look samples after chemotherapy* | | | | | | | | | | | | | | | | |
| BM | 14.10 | 1.19 | 0.12 | 0.13 | 3 | 2 | 3 | 49 | 4 | Well | Unclass | Min | No | N/A | – | 9 |
| ET | 19.40 | – | – | – | 1 | 1 | 1 | 62 | 3 | Mod | Muc | Bulk | CCIA | PR | 40 | 65 |
| DG | 6.16 | 1.02 | 0.87 | 0.02 | 4 | 2 | 1 | 70 | 3 | Mod | Endo | Min | CCIA | CR | 13 | 28 |
| FW | 1.04 | – | – | – | 4 | 1 | 1 | 52 | 3 | Mod | Serous | Bulk | CCIA | Prog | 9 | 14 |
| AB | 4.90 | 1.60 | 0.52 | 0.09 | 4 | 3 | 1 | 64 | 1 | Mod | Unclass | No | No | N/A | 18 | 59 |
| KW | 9.70 | 1.21 | 0.61 | 0.37 | 3 | 3 | 1 | 56 | 3 | poor | Endo | Min | CCIA | Pr | 28 | 30 |
| EC | 5.60 | – | – | – | 4 | 2 | 2 | 40 | 3 | Mod | Serous | Bulk | CCIA | Static | 12 | 23 |
| DG | 13.70 | 1.94 | 0.01 | 0.03 | 4 | 1 | 1 | 67 | 4 | Poor | Unclass | Min | CCIA | Static | 22 | 33 |
| DR | 7.80 | – | – | – | 4 | 1 | 1 | 72 | 3 | Poor | Endo | Min | M | Prog | 11 | 18 |
| DM | 8.40 | 1.23 | 0.04 | 0.25 | 4 | 1 | 2 | 42 | 3 | Poor | Endo | Min | CCIA | Prog | 10 | 19 |
| PS | 0.14 | 1.00 | 0.11 | 0.14 | 4 | 3 | 2 | 48 | 3 | Poor | Endo | Bulk | CCIA | Static | 10 | 19 |
| IS | 1.94 | 0.32 | 0.14 | 0.21 | 3 | 3 | 2 | 46 | 4 | Well | Endo | Min | CCIA | CR | 12 | 18 |
| NG | 1.83 | 1.07 | 0.39 | 0.12 | 4 | 3 | 1 | 65 | 3 | Poor | Unclass | Bulk | CCIA | PR | 38 | 42 |
| MD | 1.98 | 2.17 | 0.03 | 0.05 | 4 | 2 | 2 | 70 | 3 | Mod | Endo | Bulk | M | Prog | 13 | 25 |
| KD | 1.44 | 0.57 | 0.05 | 0.03 | 4 | 1 | 1 | 54 | 4 | Poor | Unclass | Bulk | CCIA | PR | 7 | 11 |
| DW | 21.10 | 1.75 | 1.75 | 0.21 | 4 | 2 | 2 | 79 | 2 | Well | Serous | No | Cyclo | Prog | 9 | 11 |
| WW | 49.40 | 0.60 | 0.09 | 0.18 | 4 | 3 | 2 | 63 | 4 | Poor | Muc | No | CCIA | CR | 7 | 12 |
| MF | 0.98 | 3.70 | 0.03 | 0.09 | 4 | 1 | 1 | 60 | 1 | Well | Serous | No | CCIA | CR | 11 | 30 |
| MP | 3.13 | 0.39 | 0.10 | 0.10 | 4 | 4 | 2 | 63 | 4 | Poor | Unclass | Bulk | CCIA | PR | 10 | 22 |
| JS | 15.30 | – | – | – | 4 | 2 | 1 | 54 | 4 | Poor | Endo | Bulk | CCIA | CR | 15 | 18 |
| JT | 4.10 | 0.77 | 0.12 | 0.34 | 3 | 2 | 2 | 47 | 3 | Poor | Serous | Bulk | CCIA | PR | 19 | 27 |
| AC | 1.63 | 1.93 | 0.16 | 0.15 | 4 | 3 | 3 | 55 | 4 | Poor | Endo | Bulk | CCIA | PR | 23 | 27 |
| MG | 94.20 | 1.03 | 0.21 | 0.39 | 3 | 2 | 1 | 63 | 4 | Poor | Endo | Min | CCIA | PR | 13 | 24 |
| ID | 78.90 | 0.23 | 0.33 | 0.11 | 3 | 2 | 1 | 47 | 4 | Mod | Serous | Min | CCIA | PR | 31 | 36 |
| JS | 3.45 | 0.02 | 0.36 | 0.41 | 2 | 2 | 1 | 68 | 3 | Mod | Endo | Min | CCIA | CR | 23 | 45 |
| ML | 11.10 | 0.39 | 0.17 | 0.06 | 4 | 3 | 2 | 44 | 2 | Well | Serous | No | CCIA | CR | – | 43 |
| IS | 6.17 | – | – | – | 3 | 3 | 2 | 46 | 4 | Well | Endo | Min | CCIA | CR | 13 | 18 |
| ES | 98.50 | 0.34 | 0.08 | 0.12 | 4 | 2 | 2 | 56 | 3 | Poor | Endo | Bulk | CCIA | PR | 14 | 19 |
| IM | 0.11 | 0.09 | 0.08 | 0.00 | 2 | 1 | 1 | 67 | 4 | Well | Serous | Bulk | M | Prog | 10 | 13 |
| LB | 22.30 | 0.40 | 0.09 | 0.01 | 4 | 2 | 1 | 34 | 3 | Poor | Serous | Bulk | CCIA | PR | 11 | 18 |
| JM | 0.12 | 1.76 | 1.17 | 0.44 | 3 | 1 | 3 | 66 | 2 | Mod | Endo | Min | CCIA | CR | – | 35 |

GST activity: nmol × $10^{-10}$ conjugate min $mg^{-1}$ protein (mean). Western blot: $\mu g$ GST 5 $\mu g^{-1}$ tumour protein. Immunohistochemistry (% cells stained positive): 1, no cells; 2, few isolated cells; 3, < 20% cells; 4, > 20% cells. Treatment (drugs used): CCIA, carboplatin, cyclophosphamide, ifosfamide, doxorubicin; M, melphalan; Carbo, carboplatin; cycle, cyclophosphamide; no, no drug treatment. Response: CR - complete response, PR - partial response, prog - progressive disease, static - no change/ < 50% reduction. Post-operative residual disease status: no, no tumour; min, < 2 cm; bulk, > 2 cm. Time to relapse and survival time in months.

**Figure 1** Western blot of protein extracts (25 μg) of seven tumour proteins (lanes 2–8) and purified GST (5 μg) in lane 1, stained for the acidic isoenzyme.

levels in ovarian biopsy samples taken prechemotherapy (first look) and those taken after chemotherapy treatment (second look), using either method of detection. This applied also to three patients for whom sequential samples were obtained. Unfortunately, because second-look procedures were not routine at this hospital only a limited number of sequential (pre- and post-chemotherapy) samples were available for analysis.

*Glutathione transferase activity*

This was measured in all 97 ovarian tumours and the results expressed as $mol^{-10}$ conjugate $min^{-1}$ $mg^{-1}$ protein. Measurements on each sample were made in triplicate and in Table II the results are detailed as the mean of the three readings. There was a wide range of values from $<10 \times 10^{-10}$ mol conjugate $min^{-1}$ $mg^{-1}$ protein to $>90 \times 10^{-1}$ mol conjugate $min^{-1}$ $mg^{-1}$ protein. There was no significant difference in GST activity in tumour samples taken prechemotherapy or post-chemotherapy, and no correlation was found between GST activity in the pre- or post-treatment tumour samples and response to treatment, progression-free or overall survival.

### Discussion

In this study two methods of detecting GST isoenzyme levels in ovarian tumour biopsies have been compared. The results obtained using immunohistochemistry and Western blot techniques were found to correlate for each of the acidic, basic and netural GST isoenzyme levels. Other methods of detecting isoenzymes have been described, including high performance liquid chromatography (HPLC) in ovarian tumours (Van Der Zee *et al.*, 1992), Western blot in breast tumours (Peters *et al.*, 1993), Western blot and Northern hybridisation in breast (Melina *et al.*, 1993). All methods of GST isoenzyme detection are subject to a degree of experimental error. In immunohistochemistry, observer variation in identifying positively stained cells may occur. In addition, infiltrating cells such as lymphocytes may also stain positively in some cases making it difficult to distinguish them from tumour cells in a stained tissue section. Semiquantitative methods (such as Western blot) involve the use of homogenised tumour tissue. This inevitably results in a degree of dilution of actual tumour protein by surrounding fatty and fibrous tissue, vascular elements and infiltrating inflammatory cells. Effort was made in this study to homogenise a piece of solid tumour by dissecting away surrounding unwanted tissue. Allowing for such experimental errors, we found that the Western blot method was more sensitive than immunohistochemistry for detecting GST isoenzyme levels in ovarian tumour biopsies. In particular, 47% of the ovarian biopsies were negative for netural GST isoenzymes and 31% were negative for basic GST isoenzymes using immunohistochemistry, whereas, a positive value was obtained for each isoenzyme in all the tumour biopsies subjected to Western blot analysis.

In the patient group studied, clinicopathological features including FIGO stage, grade of tumour differentiation, post-operative residual disease status and response to chemotherapy were found to be highly significant markers of disease outcome, as would be expected in ovarian cancer. The glutathione transferase isoenzyme levels (irrespective of the method of detection and whether the tumour sample was taken pre- or post-chemotherapy) and the GST activity were, however, not significantly correlated with response to treatment, progression-free or overall survival. This is in agreement with two previous studies in ovarian cancer using immunohistochemistry only for detection (Murphy *et al.*, 1992; Van Der Zee *et al.*, 1995) and also with another study by Van Der Zee (1992), in which GST isoenzyme levels in ovarian tumours were quantified by HPLC. However, results contrasting with our study have been found in two further immunohistochemical studies. In the first (Green *et al.*, 1993) the intensity of GST acidic isoenzyme staining was significantly correlated with survival in 78 patients with ovarian carcinoma, the prognosis being poorer in patients with a higher intensity of staining in tumour biopsies. In the second study (Hamada *et al.*, 1994), expression of GST acidic isoenzyme was again examined immunohistochemically in relation to response to chemotherapy in 61 patients with ovarian cancer. This group found that the survival of patients with positive GST acidic isoenzyme tumours was significantly shorter than those with negative tumours. The value of GST isoenzymes as markers of disease outcome have been investigated in other malignancies. In node-negative breast cancer increased GST acidic isoenzyme measured immunohistochemically was associated with decreased disease-free survival and overall survival (Gilbert *et al.*, 1993), but there was no correlation between the level of GST isoenzyme expression and the length of disease-free survival in node-positive breast cancer when the isoenzymes were quantified by Western blot (Peters *et al.*, 1993). In gastric cancer an immunohistochemical study showed no significant correlation between GST acidic isoenzyme expression and clinicopathological features or prognosis (Okuyama *et al.*, 1994), but in oral cancer the acidic isoenzyme was considered to be a useful aid to early diagnosis, prediction of disease extent and outcome (Hirata *et al.*, 1992). Immunohistochemical measurements of neutral isoenzymes have shown a positive correlation with survival in childhood acute lymphoblastic leukaemia (Hall *et al.*, 1994), and of acidic isoenzymes with survival in non-lymphoblastic leukaemia in adults (Koberda and Hellman, 1994).

A further finding from this study was that there was no significant relationship between the levels of GST isoenzymes detected in ovarian tumour biopsy samples obtained at initial surgery (before chemotherapy) and those obtained at second-look procedures (after chemotherapy). Similarly, no relationship existed between the GST activity measured in ovarian tumour samples before and after chemotherapy. Earlier *in vitro* studies in cell lines exhibiting resistance to platinum and/or alkylating agents have shown an increased enzymatic activity of GST (Lewis *et al.*, 1988; Meijer *et al.*, 1990) suggesting that repeated exposure to such drugs might result in overexpression of GST. However a study of biopsy material showed GST acidic levels to be lower in patients receiving cyclophosphamide and platinum in comparison with untreated patients (Van Der Zee *et al.*, 1992). In the current study a relationship was observed between immunohistochemically detected acidic GST isoenzyme and the treatment group comprising combination chemotherapy (carboplatin, cyclophosphamide, ifosfamide and doxorubicin). Approximately equal numbers of patients in this group had the biopsy sample taken before chemotherapy ($n = 31$) and after chemotherapy ($n = 26$). The level of GST acidic isoenzymes in prechemotherapy samples was not used as a means of selecting the treatment regimen for these patients. This measurement is, therefore, largely irrelevant–particularly when considering that the GST levels in the prechemotherapy group of patients bore no relationship to patient outcome or survival, or to any of the well-recognised clinicopathological features of ovarian cancer. When considered alone, the post-chemotherapy samples ($n = 26$) did not correlate significantly with the type of chemotherapy the patient had been treated with. Our data did not, therefore, substantiate earlier findings of any relationship between the GST isoenzyme level and treatment received.

The results of this study have shown that GST isoenzyme levels and/or GST activity cannot be used as reliable markers of disease outcome or survival in patients with ovarian cancer. However, the Western blot technique of determining GST isoenzyme levels has been demonstrated to be more sensitive than immunohistochemistry. It would, therefore, seem appropriate to suggest that studies in ovarian cancer where GST isoenzymes have shown positive correlations with survival using immunohistochemistry should be repeated employing a semiquantitative method of determining isoenzyme levels.

**Acknowledgements**

This work was supported by grants from the Christie Hospital NHS Trust and the Cancer Research Campaign.

### References

GILBERT L, ELWOOD LJ, MERINO M, MASOOD S, BARNES R, STEINBERG SM, LAZAROUS DF, PIERCE L, D'ANGELO T, MOSCOW JA, TOWNSEND AJ AND COWAN KH. (1993). A pilot study of pi-class clutathione S-transferase expression in breast cancer: correlation with estrogen receptor expression and prognosis in node-negative breast cancer. *J. Clin. Oncol.*, **11**, 49–58.

GREEN JA, ROBERTSON LJ AND CLARK AH. (1993). Glutathione-S-transferase expression in benign and malignant ovarian tumours. *Br. J. Cancer*, **68**, 235–239.

HABIG WH, PABST MJ AND JAKOBY WB. (1974). Glutathione S-transferases. The first step in mercapturic acid formation. *J. Biol. Chem.*, **249**, 7130–7139.

HALL A, AUTZEN P, CATTAN A, MALCOLM A, COLE M, KERNAHAM J AND REID M. (1994). Expression of $\mu$Class glutathione S-transferase correlates with event-free survival in childhood acute lymphoblastic leukaemia. *Cancer Res.*, **54**, 5251–5254.

HAMADA SI, KAMADA M, FURUMOTO H, HIRAO T AND AONO T. (1994). Expression of Glutathione-S-Transferase-$\pi$ in human ovarian cancer as an indicator or resistance to chemotherapy. *Gynecol. Oncol.*, **52**, 313–319.

HIRATA S, ODAJIMI T, KOHAMA G, ISHIGAKI S AND NIITSU Y. (1992). Significance of Glutathione S-Transferase–$\pi$ as a tumour marker in patients with oral cancer. *Cancer*, **70**, 2381–2387.

KAPLAN E AND MEIER P. (1958). Non-parametric estimation from incomplete observations. *J. Am. Stat. Assoc.*, **5**, 457–481.

KOBERDA J AND HELLMAN A. (1991). Glutathione-S-transferase activity of leukaemia cells as a prognostic factor for response to chemotherapy in acute leukaemias. *Med. Oncol. Tumour Pharmacother.*, **8**, 35–38.

LAEMMLI VK. (1970). Cleavage of structural proteins during the assembly of the head of bacteriaphage T4. *Nature*, **227**, 680–685.

LEWIS AD, HAYES JD AND WOLF RD. (1988). Glutathione and glutathione-dependent enzymes in ovarian adenocarcinoma cell lines derived from a patient before and after the onset of drug-resistance: intrinsic differences and cell cycle effect. *Carcinogenesis*, **9**, 1283.

MANNERVIK B AND DANIELSON UH. (1988). Glutathione transferase–structure and catalytic activity. *Critical Rev. Biochem.*, **23**, 283.

MEIJER C, MULDER NH AND DE VRIES EGE. (1990). The role of detoxifying systems in resistance of tumour cells to cisplatin and adriamycin. *Cancer Treat. Rev.*, **17**, 389.

MELINA R, OESTEREICH S, ZHOU JL, TANDON AK, CLARK GM, ALLRED DC, TOWNSEND AJ, MOSCOW AJ, COWAN KH, MCGUIRE WL AND FUQUA SAW. (1993). Glutathione Transferase GST $\pi$ in breast tumours evaluated by three techniques. *Dis. Markers*, **11**, 71–82.

MURPHY D, MCGOWN AT, HALL A, CATTAN A, CROWTHER D AND FOX B. (1992). Glutathione-S-transferase activity and isoenzyme distribution in ovarian tumour biopsies taken before or after cytotoxic chemotherapy. *Br. J. Cancer*, **66**, 937–942.

OKUYAMA T, MAEHARA Y, ENDO K, BABA H, ADACHI Y, OKUWANO M AND SUGIMACHI K. (1994). Expression of Glutathione-S-transferase-pi and sensitivity of human genetic cancer cells to aspiatin. *Cancer*, **74**, 1230–1236.

PETERS WHM, ROELOFS HMJ, VAN PUTTEN WLJ, JANSEN JBMJ, KLIJN JGM AND FOEKENS JA. (1993). Response to adjuvant chemotherapy in primary breast cancer: no correlation with expression of glutathione-S-transferases. *Br. J. Cancer*, **68**, 86–92.

TIDEFELT U, ELMHORN-ROSENBORG A, PAUL C, HAO X-Y, MANNERVIK B AND ERIKSSON L. (1992). Expression of Glutathione Transferase $\pi$ as a predictor for treatment results at different stages of acute nonlymphoblastic leukaemia. *Cancer Res.*, **52**, 3281–3285.

VAN DER ZEE AGJ, OMMEN B, MEIJER C, HOLLEMA M, VAN BLADEREN PJ AND DE VRIES EGE. (1992). Glutathione S-transferase activity and isoenzyme composition in benign ovarian tumours, untreated ovarian tumours, and malignant ovarian tumours after platinum/cyclophosphamide chemotherapy. *Br. J. Cancer.*, **66**, 930–936.

VAN DER ZEE AGJ, HOLLEMA H, SUURMEIJER AJH, KRANS M, SLUITER WJ, WILLEMSE PHB, AALDERS JG AND DE VRIES EGE. (1995). Value of $\rho$-Glycoprotein, Glutathione-S-transferase pi, C-erbB-2 and $\rho$53 as prognostic factors in ovarian carcinomas. *J. Clin. Oncol.*, **13**, 70–78.